# PReS-FINAL-2288: Juvenile primary and secondary antiphospolipid syndrome. Clinical and serological features on a Colombian cohort

**DOI:** 10.1186/1546-0096-11-S2-P278

**Published:** 2013-12-05

**Authors:** C Malagon, AC Mosquera, MDP Gomez, C Vargas

**Affiliations:** 1 Universidad El Bosque, Bogota, Colombia

## Introduction

Antiphospholipid syndrome (APS) is characterized by venous or arterial thrombosis and/or fetal losses, in the presence of one or more types of antiphospholipid antibodies (aPL) (Anitcardiolipin antibodies (ACA), lupus anticoagulant (LA) or anti beta 2-glycoprotein I (β2GPI). Information about APS in juvenile patients is limited and reports showed some differences between the clinical and serological features in adults and juvenile APS. There are limited data on the incidence and prevalence of Primary APS (PAPS) and Secondary APS (SAPS). Thrombotic events are the typical manifestation of APS but hematologic and neurologic manifestations have been described with different frequencies.

## Objectives

To describe a group of juvenile patients with APS and to compare the clinical and serological manifestations of PAPS and SAPS.

## Methods

This is a descriptive case series study. Includes patients from three pediatric rheumatology clinics in Bogota and Cali, Colombia.

## Results

There were 69 APS juvenile patients. Sex ratio: F 5.9: M 1. Mean age at onset: 12.3 years (2-17 years). Mean follow up 30 months (5-120). Antinuclear antibodies were positive in 83%, IgG ACA 73%, IgM ACA 74% and LA 58%. Anti β2GPI was not measured in all patients. 41% developed one or more thrombotic events, 93% developed thrombotic and no thrombotic manifestations and 58% had two or more non thrombotic manifestations. 28/69 patients developed 44 thrombotic events during follow up. Two juvenile Systemic Lupus Erythematous (jSLE) patients developed catastrophic APS. Deep venous thrombosis (DVT) was more common in PAPS and arterial thrombosis was more frequent on SAPS without statistical significance (p 0.209 and 0.299). Arterial thrombosis was documented on 16 patients (pulmonary thromboembolism, cerebrovascular events, peripheral arterial thrombosis and bone and liver infarcts) but there were not significant differences on frequency and type of thrombosis between PAPS and SAPS. 29% had recurrences of thrombosis without significant differences between PAPS and SAPS (p value 0.134).

Thrombocytopenia was the most common non thrombotic manifestation and was more frequent on PAPS while hemolytic anemia was more common SAPS (p values 0, 05 and 0,058). Neurological complications had a similar frequency on both groups. Raynaud and livedo reticularis were more common on SAPS but p values were not significant. Auto antibodies profile were also very similar on PAPS and SAPS. All types of aPL were detected on PAPS and SAPS with a similar frequency on patients with and without thrombosis.

## Conclusion

APS may determine an important morbidity. Is a frequent cause on thrombosis in juvenile patients who may have or not an auto immune disease like jSLE. Non thrombotic symptoms are very common on PAPS and SAPS. Hematologic, neurological and skin manifestations are frequent on both groups. All types of aPL were detected on PAPS and SAPS and the frequency was similar on patients who developed thrombosis and who do not. Screening of aPL on patients with thrombosis and hematological, neurological and dermatological complications as described must be performed on a regular basis and may allow a better follow up and prevent complications.

## Disclosure of interest

None declared.

